# Primary gastric epithelioid angiosarcoma: A case report of diagnostic biopsy pitfall

**DOI:** 10.1097/MD.0000000000039719

**Published:** 2024-10-04

**Authors:** Ning Zhou, Li Yang, Tao Li, Liqiao Chen, Ruiqi Jia, Ying Chen

**Affiliations:** a Department of Pathology, Mianyang 404 Hospital, Mianyang, Sichuan Province, China; b Department of Pathology, Santai County People’s Hospital, Mianyang, Sichuan Province, China; c Department of Pathology, Guiqian International General Hospital, Guiyang, Guizhou Province, China.

**Keywords:** adenocarcinoma, epithelioid angiosarcoma, gastric angiosarcoma, immunohistochemistry, stomach

## Abstract

**Rationale:**

Angiosarcoma is an aggressive neoplasm derived from endothelial cells that may develop anywhere within the body. Here, we report a case of primary gastric epithelioid angiosarcoma initially misdiagnosed as poorly differentiated adenocarcinoma from the preoperative biopsy.

**Patient concerns:**

The patient was a 60-year-old male admitted to the hospital due to abdominal discomfort. The gastroscopy examination suggested advanced gastric cardia carcinoma. Subsequent biopsy pathology examination confirmed the diagnosis of poorly differentiated adenocarcinoma. Therefore, the patient underwent radical resection for proximal gastric cancer. Histopathology showed that the tumor cells were epithelioid with rich, eosinophilic cytoplasm. Immunohistochemical examination indicated that the tumor cells were CD34, CD31, and ERG.

**Diagnoses:**

Based on clinical, morphological, and immunophenotypic evidence, primary gastric epithelioid angiosarcoma diagnosis was confirmed.

**Interventions:**

The postoperative Tumor Node Metastasis staging was considered T4aN1Mx. The patient did not receive chemotherapy.

**Outcomes:**

The patient died 3 months after the operation.

**Lessons:**

Primary gastric epithelioid angiosarcoma is a rare gastric tumor. Given the epithelioid cell features displayed by tumor cells and the high expression of epithelial markers in tumor cells (83%), preoperative diagnosis is difficult and should be differentiated from adenocarcinoma or gastrointestinal stromal tumor.

## 1. Introduction

Angiosarcoma is a rare soft tissue tumor that accounts for 1% to 2% of all soft tissue sarcomas.^[1]^ It can develop anywhere in the body. Two-thirds of such tumors are cutaneous, mainly found in the head and neck.^[2]^ They vary from highly differentiated tumors resembling a hemangioma to anaplastic lesions difficult to distinguish from a poorly differentiated carcinoma or pleomorphic sarcoma. Epithelioid angiosarcoma is a unique morphologic subtype of angiosarcomas in which the malignant endothelial cells have a predominantly epithelioid appearance.^[3]^ This study aims to raise awareness of this rare and highly aggressive neoplasm and differentiate it from poorly differentiated adenocarcinomas, epithelioid gastrointestinal stromal tumors (GISTs).

## 2. Case presentation

### 2.1. Case characteristics

**Figure 1.**
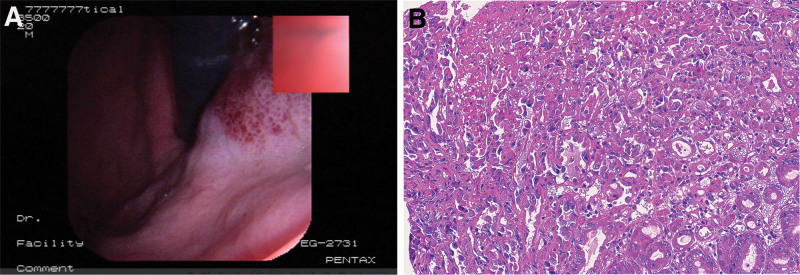
Gastroscopy revealed a polypoid neoplasm located on the lesser curvature of the cardia (A);the tumors cells were epithelioid with rich, eosinophilic cytoplasm and the histological morphology resembled that of poorly differentiated adenocarcinomas (B, ×400).

The patient was a 60-year-old male hospitalized because of pain and discomfort in the abdomen. The patient has no special family history and the physical examination did not reveal any significant abnormalities. Herhemoglobin level was 105 g/L. The platelet count, coagulation function, and blood biochemistry were within normal limits. Abdominal ultrasound showed no abnormal ultrasound images in the liver, gallbladder, pancreas, and spleen. CT scan of the lungs showed chronic bronchitis and emphysema. Gastroscopy revealed a polypoid neoplasm located on the lesser curvature of the cardia, featuring a turbid uneven surface and brittle texture (Fig. 1A), suggestive of advanced gastric cardia carcinoma. In a subsequent biopsy, the tumors cells were epithelioid with rich, eosinophilic cytoplasm and it was pathologically diagnosed as poorly differentiated adenocarcinoma (Fig. 1B). The tumor was palpable during the surgery, presenting a hard consistency. The lesion center was situated on the lesser curvature of the cardia, infiltrating the serous layer; neither noticeable metastasis in the liver and spleen nor ascites was evident. No discernible metastatic nodules were identified in the pelvic cavity, mesentery, or abdominal wall. The patient underwent radical surgery for proximal gastric cancer.

### 2.2. Pathological findings

**Figure 2.**
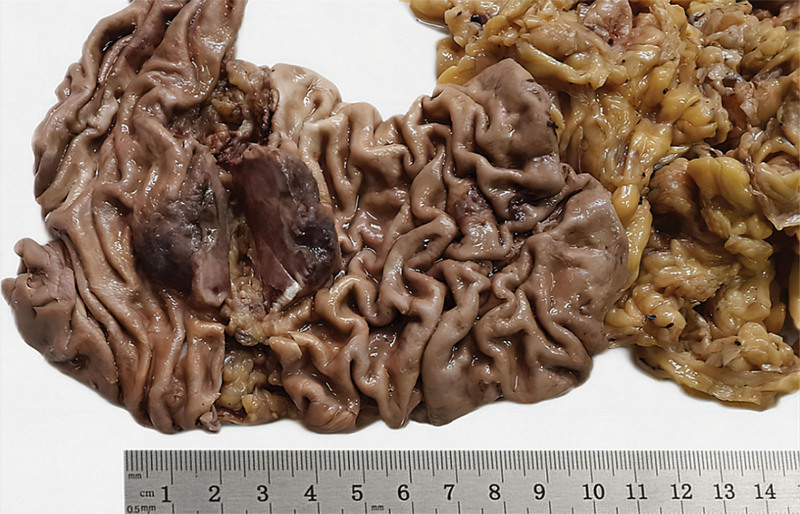
The polypoid tumor with dark brown cross-section and visible surface ulcer.

**Figure 3.**
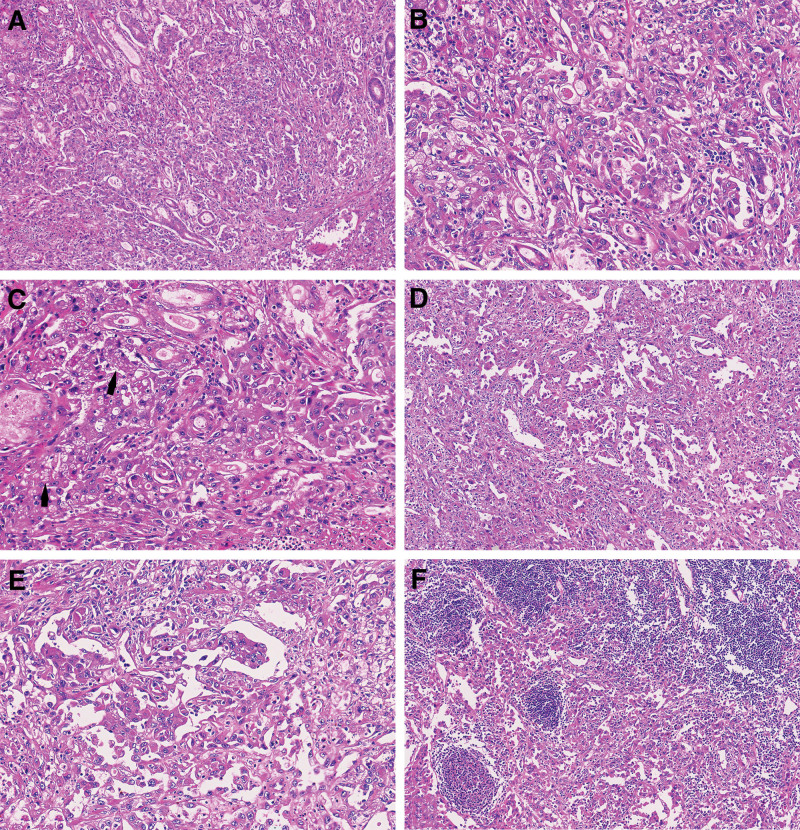
H&E staining results: the tumor cells showed infiltrative growth within the gastric mucosa (A, ×100); local magnification of A, the tumor cells appeared epithelioid, cytoplasm-rich, eosinophilic, with damaged and residual mucosal glands and morphological resemblance to poorly differentiated carcinoma (B, ×400); local magnification of A, multiple small vacuoles could be observed in the cytoplasm of tumor cells as indicated by the arrow (C, ×400); interlaced, incomplete vascular networks were dispersed in the gastric wall tissue (D, ×200); epithelioid tumor cells grew and accumulated in multiple layers on the luminal surface, or formed clustered and papillary projections towards the lumen (E, ×400); lymph node metastasis (F, ×200).

**Figure 4.**
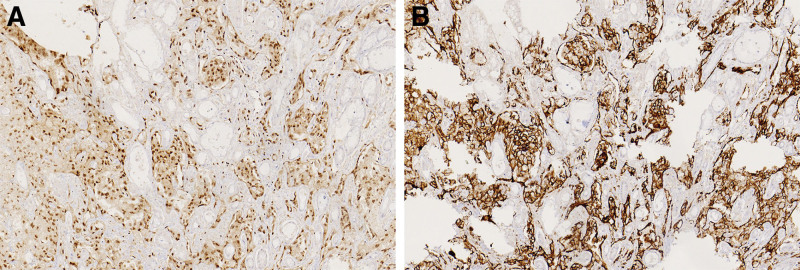
Immunohistochemical staining results: nucleus positive for ERG (A, ×200); membrane positive for CD31 (B, ×200).

Grossly, for part of the resected stomach and omentum specimens, a polypoid tumor measuring 3.5 cm × 3 cm × 1.5 cm was visible at the cardia. Its surface was ulcerated, and the cross-section manifested as dark red with visible bleeding (Fig. 2). Upon microscopic inspection, the tumor cells exhibited arrangement in small clusters or sheet-like structures. The cells were epithelioid with rich, eosinophilic cytoplasm (Fig 3A and B). The chromatin appeared vacuolated, the nucleus was large, and nucleoli were visible. Obvious nuclear atypia with nuclear divisions of roughly 3/10 HPF was observed, and some cells contained one or several small vacuoles of various sizes in the cytoplasm (Fig. 3C). In certain regions, a fine and incomplete network of blood vessels was observed interweaving in the gastric wall tissue, dividing and encompassing the surrounding muscle and nerve tissue (Fig. 3D). Tumor cells were seen growing into multiple layers along the luminal surface or forming clusters or papillary protrusions toward the luminal surface (Fig. 3E). In some areas, vascular-like lacunae were clustered, lined with a single layer of thin neoplastic endothelial cells, resembling granulation tissue. Many red blood cells were observed in the lumen. Extensive hemorrhage and necrosis occurred in the stroma. Immunohistochemical examinations showed that the tumor cells were positive for ERG (Fig. 4A), CD34, CD31 (Fig. 4B), and vimentin, whereas CK (AE1/AE3), EMA, CK7, HMB45, SMA, CD117, Dog-1, and S-100 were negative; Ki-67 labeling index was 30%. The tumor cells infiltrated the serous layer, and perigastric lymph node metastasis in 3/10 (Fig. 3F). The postoperative Tumor Node Metastasis staging was considered T4aN1M0.

### 2.3. Diagnosis

Based on clinical, morphological, and immunophenotypic evidence, primary gastric epithelioid angiosarcoma diagnosis was confirmed. The patient did not receive chemotherapy and died 3 months after surgery due to lung metastasis.

Angiosarcoma is a malignant soft tissue tumor that arises from vascular and lymphatic endothelial cells. These tumors are known for their rapid proliferation, aggressive infiltration, hematogenous metastasis, and relatively poor prognosis with low life expectancy. The exact pathogenesis remains unclear; however, various long-term chronic stimuli, such as chronic lymphedema, chronic venous ulcers, exposure to radiation, and long-term exposure to substances such as vinyl chloride, thorium dioxide, and arsenic, may act as contributing factors.^[4,5]^ Particularly, chronic venous ulceration may become an independent risk factor in future research.^[6]^ Typically, these tumors occur in the superficial soft tissues of the skin and subcutaneous tissues and less frequently in parenchymal organs like the liver, spleen, adrenal gland, ovary, heart, lung, and breast. Incidences in the gastrointestinal tract are rare.^[7]^ Primary tumors have been reported in the esophagus, stomach, small intestine, colon, rectum, and appendix, with the small intestine being the most common site.^[8]^

Angiosarcomas typically show significant histological changes. Highly differentiated angiosarcomas may reveal an interconnected network or sinusoidal structure of well-differentiated blood vessels, which can easily lead to misdiagnosis as benign vascular tumors. However, angiosarcomas are distinguishable from benign vascular tumors by their incomplete vascular lumina with irregular size and shape, disorganized structure, and infiltrative growth into surrounding tissues. Endothelial cells typically show mild to moderate atypia, growing and accumulating along the luminal surface or forming papillary protrusions into the lumen, with frequent mitotic divisions. Poorly differentiated angiosarcomas exhibit more pronounced atypia, with tumor cells arranged in solid sheets or nest-like structures, and cells can be epithelioid or spindle-shaped. These tumor cells display significant nuclear atypia with large nuclei, prominent nucleoli, frequent mitotic division, and frequent extensive tumor necrosis in the stroma. When the tumor is predominantly composed of epithelioid cells, it is termed epithelioid angiosarcoma. Such tumors are morphologically similar to poorly differentiated carcinoma.

Primary gastric epithelioid angiosarcoma is exceedingly rare. The first case was reported by Amy C et al in 2001.^[8]^To the best of our knowledge, only 5 cases of primary epithelioid angiosarcomas of the stomach have been reported in the English literature,^[4,8–11]^ as indicated in Table 1. All 6 patients were middle-aged and older individuals ranging from 56 to 84 years old, with an average age of 70. Males slightly outnumbered females (4:2). The clinical manifestations lacked specificity, with the main symptoms including melena, fatigue, abdominal discomfort, and anemia; no clear cause was identified in any of the patients. Tumors of 3 patients were located in the gastric body, one in the pylorus, one in the cardia, and 2 were multifocal lesions in the stomach and intestine. Most patients presented with polypoid masses with visible surface bleeding and ulceration. Epithelioid angiosarcoma is characterized by an extremely aggressive course, leading to very poor prognoses. At the time of the hospital visit, 3 patients had lymph nodes, one of whom also had bone metastases. All patients died within 8 months following the surgery.

**Table 1:** All reported cases of gastric epithelioid angiosarcoma.

Study (year)	Age (sex)	Clinical presentation	Preoperative pathological diagnosis	Immunohistochemical	Tumour site (tumor size)	Metastatic sites	Survival data
Amy C et al (2001)^[8]^	70 (male)	N/A	Poorly differentiated adenocarcinoma	Positive: cytokeratin and vascular antigens	N/A	N/A	8 months
Jie Xia et al (2018)^[4]^	56 (male)	Melena and epigastric dull pain	Malignant tumor	Positive: CK (AE1/AE3), CD31, ERG, FLI-1, EMA, CAM5.2, CK7, and Vimentin Negative: EBER, HCG, c-Myc, P63, D2–40, c-erbB-2, PLAP, CD68, MelanA, HMB45, Desmin, SMA, DOG1, CD117 and S100	Fundus (7 cm)	NO	2 months
Chen YW et al (2020)^[9]^	77 (female)	Melena, dizziness and anemia	Inflammatory infiltration in the gastric mucosa	Positive: CK(AE1/AE3), CD31, CD34, EMA, vimentin Negative: S100, CD117, DOG1, CD56, SYN, CgA, LCA, desmi, ALK	Polypoid lesion in the gastric body, multiple polypoid lesions in the ileocecum, ascending colon, transverse colon and sigmoid colon	Lymphatic and osseous metastasis	3 months
Artem Sharko et al (2021)^[10]^	84 (female)	Fatigue, shortness of breath, melena and anemia	NA	Positive: AE1/AE3 and CK8/18, CD31, ERG, FLI1, vimentin Negative: LANA, D2–40, Melan A, SOX10, S100, HMB45, MITF, SMA, CD117 and DOG1	Polyp in the pyloric channel (0.7 cm) Polyp in the fundus (1.2 cm)	NO	N/A
Yu JH et al (2023)^[11]^	75 (male)	Fatigue, melena, dysuria and anemia	Poorly differentiated gastric adenocarcinoma	Positive: CK7, CD31, ERG, cd34, vimentin, B-catenin, P53 Negative: CK20, CDX-2, CEA, SMA, DOG-1, CD117, LCA, Her-2 (0)	Irregular mass in the fundus (2.0 cm) Multiple nodular lesions in the jejunum (0.5–2.0 cm)	Lymph node metastasis	2 months
The present case	60 (male)	Cardia	Poorly differentiated gastric adenocarcinoma	Positive: CD31, ERG, CD34, vimentin Negative: CK(AE1/AE3), EMA, CK7, HMB45, SMA, CD117, DOG-1, and S-100	Polyploid lesion in the cardia (3.5 cm)	Lymph node metastasis	3 months

Microscopically, the tumor cells mainly formed solid nest-, cluster-, or sheet-like structures with abundant cytoplasm and an epithelioid appearance. The histological morphology resembled that of poorly differentiated carcinomas. Immunohistochemical study is the best way to accurately diagnose epithelioid angiosarcomas. These cells express endothelial cell-associated markers ERG, CD31, CD34, and factor VIII-related antigens. Among these, ERG and CD31 exhibit high sensitivity and specificity, with the sensitivity for angiosarcoma approaching 100%. A confident diagnosis of angiosarcoma can be made when ERG and CD31 are both positive.^[12]^ A noteworthy pitfall is that gastric epithelioid angiosarcoma nearly always expresses one or more epithelial markers. Out of 6 primary gastric epithelioid angiosarcoma cases, 5 (83%) expressed epithelial markers, including CK (AE1/AE3), CK7, and EMA, among others. Three (50%) of these cases were initially misdiagnosed as poorly differentiated adenocarcinomas by pathologists during preoperative biopsies.

Due to the rarity of primary gastric epithelioid angiosarcoma and its unique histological and immunohistochemical features, histologic diagnosis of gastric epithelioid angiosarcomas has always been challenging for pathologists. Particularly, pathologists should consider differentiating this tumor from other tumors with epithelioid morphology, such as poorly differentiated carcinoma and epithelioid GISTs, to avoid misdiagnosis in endoscopic biopsy samples. Angiosarcomas generally appear as purple-brown or dark brown upon sectioning. Apart from the solid, sheet-like, or small cluster epithelioid cell areas, tumor cells can also be observed forming vascular areas. Patches of tumor cells connect to form reticular structures with red blood cells inside. Vacuoles of various sizes can be seen within the cytoplasm of the tumor cells, occasionally with one or more red blood cells inside, which indicates a vascular tumor. Poorly differentiated adenocarcinomas and epithelioid GISTs do not express characteristic vascular markers such as CD31 and ERG, with the latter expressing CD117 and Dog-1, allowing differentiation.

Due to the limited number of reported cases of primary gastric epithelioid angiosarcoma, treatment guidelines for the disease have yet to be established. The prognosis for patients diagnosed with angiosarcoma is very poor, and it is determined by a number of factors. A 14-year retrospective review showed that the overall 5-year survival rate for 125 patients with angiosarcomas at various sites was 31%.^[13]^ However, patients with gastric epithelioid angiosarcomas usually die within 8 months after diagnosis (Table 1). For gastrointestinal angiosarcoma cases, complete surgical resection is considered the only factor that correlates with disease-free survival.^[14]^ Radiation therapy is usually used as adjuvant therapy after non-radical resection to reduce the postoperative recurrence rate. Chemotherapy with drugs such as paclitaxel and epirubicin can be the primary treatment method for unresectable or metastatic tumors. Reports suggest that angiosarcomas can express PD-L1, and patients have shown improved prognosis following PD-L1 inhibitor pembrolizumab treatment.^[15]^ Therefore, immunotherapy could be a promising new therapy for angiosarcomas. However, larger-scale research is needed to provide conclusive evidence regarding the efficacy of this class of drugs.

## 3. Conclusions

In summary, primary gastric epithelioid angiosarcoma is exceedingly rare. Due to the appearance of epithelioid cell morphology in this tumor cell, and the gastric epithelioid angiosarcoma is highly susceptible to the expression of epithelial markers such as CK (83%), making it easy to misdiagnose poorly differentiated adenocarcinoma in endoscopic biopsy samples. This study aims to raise awareness of this rare and highly aggressive neoplasm and differentiate it from poorly differentiated adenocarcinomas, epithelioid GISTs.

## Acknowledgments

We would like to thank Editage (www.editage.cn) for English language editing.

## Author contributions

**Conceptualization:** Ning Zhou, Ying Chen.

**Data curation:** Li Yang, Tao Li, Liqiao Chen, Ruiqi Jia.

**Methodology:** Tao Li.

**Writing – original draft:** Li Yang.
